# Estimated Risk of Adverse Surgical Outcomes Among Patients With Recent COVID-19 Infection Using Target Trial Emulation Methods

**DOI:** 10.1001/jamanetworkopen.2023.4876

**Published:** 2023-03-28

**Authors:** William J. O’Brien, Kalpana Gupta, Kamal M. F. Itani

**Affiliations:** 1 Center for Healthcare Organization and Implementation Research, Veterans Affairs Boston, Boston, Massachusetts; 2 Department of Medicine, Veterans Affairs Boston, Boston, Massachusetts; 3 Department of Surgery, Veterans Affairs Boston, Boston, Massachusetts

## Abstract

This cohort study emulates a trial within a large national veteran population to assess the risk of adverse postoperative outcomes among patients with recent COVID-19 infection.

## Introduction

The American Society of Anesthesiologists published recommendations on the timing of elective surgery for patients who have recently recovered from COVID-19.^1,2^ Several recent studies are conflicting about optimal timing of surgery relative to vaccination and infection. We emulated a trial within a large veteran population to assess the risk of adverse postoperative outcomes among patients with recent COVID-19 infection. We hypothesized that patients with infection in the 30 or 60 days before surgery would face increased risk compared with those without infection.

## Methods

This cohort study was a target trial emulation with pseudorandomization to control (without recent infection) vs 2 mutually exclusive exposures (most recent infection in preoperative days 1-30 or 31-60). Eligible patients underwent major surgery from January 1 to September 30, 2021 (Alpha and Delta strain prevalence). All participants were encouraged to receive vaccination, and policy was to test preoperatively using reverse transcription–polymerase chain reaction. No oral outpatient therapies were in use. The Veterans Affairs Boston institutional review board approved this study and approved a waiver of informed consent, as the risk to participants was considered minimal. The STROBE reporting guideline was followed.

Time zero for pseudorandomization was preoperative day 60. A propensity score model and stabilized inverse probability of treatment weighting balanced characteristics across exposure groups. Propensity score covariates were factors known at time zero that would be associated with exposure and were considered balanced if standardized mean differences were less than 0.2. We did not assess infection severity.

The end point was death, cardiac events, central nervous system outcomes, respiratory outcomes, surgical infection, or thromboembolic events within 30 days after surgery. Weighted logistic regression estimated the odds ratio (OR) of any outcome as a function of exposure group, perioperative factors (because they were not known at time zero), and any propensity score covariates unbalanced after weighting. The eMethods in Supplement 1 contain greater detail.

## Results

A total of 29 093 patients (mean [SD] age, 66.1 [11.4] years; 90.0% men; 67.5% White patients) underwent surgery at 123 hospitals (Table 1). Among the 15 553 surgical procedures (53.5%) performed for inpatients, the mean (SD) length of stay was 5.0 (8.5) days. The median time between infection and surgery was 30 days (IQR, 13-44 days). Adverse postoperative outcomes occurred among 1337 of 28 635 patients (4.7%) within the group without COVID-19, 18 of 238 (7.6%) within the group with a 1- to 30-day infection, and 7 of 220 (3.2%) within the group with a 31- to 60-day infection. The ORs for postoperative outcomes were 1.40 (95% CI, 0.77-2.35) among those with infection in preoperative days 1 to 30 and 0.68 (95% CI, 0.26-1.42) among those with infection in preoperative days 31 to 60 (Table 2).

**Table 1.** Baseline Characteristics of Surgical Patients Stratified by Prior COVID-19 Infection

Characteristic	Patients, No. (%)								
	Overall	Observed cohort				Balanced cohort			
		Without COVID	COVID days 1-30 prior^a^	COVID days 31-60 prior^a^	SMD	Without COVID	COVID days 1-30 prior^a^	COVID days 31-60 prior^a^	SMD
No. surgeries	29 093	28 635	238	220	NA	28 633.6	219.4	193.6	NA
**Propensity score covariates** ^b^									
Aged ≥65 y	18 324 (63.0)	18 049 (63.0)	140 (58.8)	135 (61.4)	0.058	18 035.2 (63.0)	128.7 (58.7)	122.6 (63.3)	0.064
Male	26456 (90.9)	26 028 (90.9)	224 (94.1)	204 (92.7)	0.082	26 038.4 (90.9)	205.5 (93.7)	179.6 (92.8)	0.069
Race									
Non-White	8035 (27.6)	7902 (27.6)	69 (29.0)	64 (29.1)	0.086	7907.4 (27.6)	59.1 (26.9)	49.6 (25.6)	0.098
Not known	1406 (4.8)	1392 (4.9)	6 (2.5)	8 (3.6)		1383.8 (4.8)	5.2 (2.4)	6.0 (3.1)	
White	19 652 (67.5)	19 341 (67.5)	163 (68.5)	148 (67.3)		19 342.4 (67.6)	155.1 (70.7)	138.0 (71.3)	
ASA classification									
1-2	4178 (14.4)	4122 (14.4)	30 (12.6)	26 (11.8)	0.051	4112.0 (14.4)	29.5 (13.5)	23.2 (12.0)	0.046
3-5	24915 (85.6)	24 513 (85.6)	208 (87.4)	194 (88.2)		24 521.6 (85.6)	189.8 (86.5)	170.3 (88.0)	
BMI ≥35	4819 (16.6)	4741 (16.6)	39 (16.4)	39 (17.7)	0.024	4742.2 (16.6)	35.8 (16.3)	30.3 (15.6)	0.017
Smoking	7105 (24.4)	7013 (24.5)	49 (20.6)	43 (19.5)	0.080	6992.4 (24.4)	48.3 (22.0)	44.5 (23.0)	0.038
Diabetes	8196 (28.2)	8039 (28.1)	81 (34.0)	76 (34.5)	0.093	8065.8 (28.2)	65.1 (29.7)	57.4 (29.7)	0.022
Serum albumin ≥3.5 g/dL	17221 (59.2)	16 978 (59.3)	126 (52.9)	117 (53.2)	0.085	16 949.8 (59.2)	123.1 (56.1)	110.6 (57.1)	0.041
COVID-19 vaccine status^c^									
None	15 706 (54.0)	15 338 (53.6)	179 (75.2)	189 (85.9)	0.515	15 457.1 (54.0)	129.7 (59.1)	109.3 (56.5)	0.082
Partial	2305 (7.9)	2283 (8.0)	17 (7.1)	5 (2.3)		2268.6 (7.9)	18.3 (8.3)	14.3 (7.4)	
Full	11 082 (38.1)	11 014 (38.5)	42 (17.6)	26 (11.8)		10 907.9 (38.1)	71.4 (32.5)	70.0 (36.2)	
Geographic region									
Continental^d^	3809 (13.1)	3746 (13.1)	28 (11.8)	35 (15.9)	0.234	3748.5 (13.1)	30.6 (13.9)	27.4 (14.1)	0.110
Midwest	6974 (24.0)	6847 (23.9)	60 (25.2)	67 (30.5)		6863.7 (24.0)	58.0 (26.4)	48.1 (24.9)	
North Atlantic	7270 (25.0)	7181 (25.1)	52 (21.8)	37 (16.8)		7155.7 (25.0)	53.1 (24.2)	41.7 (21.5)	
Pacific	5342 (18.4)	5266 (18.4)	35 (14.7)	41 (18.6)		5257.7 (18.4)	31.9 (14.5)	37.2 (19.2)	
Southeast	5698 (19.6)	5595 (19.5)	63 (26.5)	40 (18.2)		5608.0 (19.6)	45.8 (20.9)	39.2 (20.3)	
**Outcome model covariates**									
Emergency surgery	1183 (4.1)	1160 (4.1)	12 (5.0)	11 (5.0)	0.032	1161.5 (4.1)	14.0 (6.4)	5.7 (2.9)	0.109
Operative duration	2.10 (1.60)	2.10 (1.61)	1.84 (1.20)	2.09 (1.61)	0.122	2.10 (1.61)	1.83 (1.17)	2.00 (1.52)	0.128
Surgical specialty									
Orthopedics	8312 (28.6)	8195 (28.6)	60 (25.2)	57 (25.9)	0.314	8192.5 (28.6)	60.3 (27.5)	51.3 (26.5)	0.309
Otolaryngology	814 (2.8)	803 (2.8)	7 (2.9)	4 (1.8)		802.4 (2.8)	6.0 (2.7)	5.1 (2.6)	
General surgery	7137 (24.5)	7024 (24.5)	51 (21.4)	62 (28.2)		7022.8 (24.5)	44.9 (20.5)	54.7 (28.3)	
Neurosurgery	1569 (5.4)	1553 (5.4)	10 (4.2)	6 (2.7)		1553.3 (5.4)	8.5 (3.9)	3.1 (1.6)	
Obstetrics and gynecology	371 (1.3)	365 (1.3)	2 (0.8)	4 (1.8)		363.6 (1.3)	2.1 (1.0)	2.8 (1.4)	
Peripheral vascular	4060 (14.0)	3977 (13.9)	54 (22.7)	29 (13.2)		3982.3 (13.9)	48.1 (21.9)	21.9 (11.3)	
Plastic surgery	561 (1.9)	547 (1.9)	7 (2.9)	7 (3.2)		546.5 (1.9)	5.6 (2.5)	4.8 (2.5)	
Podiatry	686 (2.4)	669 (2.3)	12 (5.0)	5 (2.3)		671.0 (2.3)	8.6 (3.9)	5.4 (2.8)	
Thoracic surgery	639 (2.2)	627 (2.2)	8 (3.4)	4 (1.8)		626.4 (2.2)	7.2 (3.3)	5.2 (2.7)	
Urology	4944 (17.0)	4875 (17.0)	27 (11.3)	42 (19.1)		4872.8 (17.0)	28.1 (12.8)	39.2 (20.2)	

Abbreviations: ASA, American Society of Anesthesiologists; BMI, body mass index (calculated as weight in kilograms divided by height in meters squared); NA, not applicable; SMD, standardized mean difference.

SI conversion factor: To convert albumin to grams per liter, multiply by 10.0.

^a^ The “prior” in the column headings is relative to the date of surgery.

^b^ Factors known at time zero that would be associated with exposure and were considered balanced if SMDs were less than 0.2.

^c^ COVID-19 vaccine status is on day 60 prior to surgery.

^d^ Includes Montana, Wyoming, Louisiana, Texas, Missouri, Colorado, Arkansas, Oklahoma, and Utah.

**Table 2.** Weighted Logistic Regression for Outcome of Any Adverse Postoperative Outcome^a^

Covariate	Odds Ratio (95% CI)
Main exposure (3 levels)	
Without prior COVID-19	1 [Reference]
COVID-19	
Days 1-30 prior	1.40 (0.77-2.35)
Days 31-60 prior	0.68 (0.26-1.42)
Perioperative factors^b^	
Emergency vs elective surgery	3.42 (2.83-4.10)
General anesthesia vs other	1.23 (1.02-1.49)
Operative duration, h	1.28 (1.25-1.31)

^a^ Any adverse postoperative outcome consists of 30-day mortality, cardiac outcomes, central nervous system outcomes, respiratory outcomes, infectious diseases outcomes, or thromboembolic outcomes.

^b^ Emergency surgery, anesthesia, operative duration, and surgical specialty were included as postrandomization confounders; surgical specialty was omitted.

## Discussion

These findings suggest that recent COVID-19 infection was not associated with risk of adverse postoperative outcomes, regardless of timing within the previous 60 days. A recent study found surgery within 8 weeks after a positive COVID-19 test result was associated with higher 90-day mortality vs matched controls.^3^ Another study found lower perioperative risk for vaccinated patients and for unvaccinated patients not given general anesthesia.^4^ These studies may shift the timing of surgery relative to recent infection. Our study further rebalances the scale in favor of performing surgery in recently recovered patients.

Study strengths include a national surgical registry and reliable assessment of vaccination and infection. The target trial emulation was chosen to reduce certain biases.^5^ However, some bias will remain due to unmeasured covariates and a selection bias related to patients who did not undergo surgery due to clinical status. Nevertheless, current evidence suggests the decision to proceed should be based on clinical expertise rather than a fixed time interval after infection, consistent with current guidelines.

## Notes

Supplement 1.
eMethods.

Supplement 2.
Data Sharing Statement
