# Supplementary material for: Estimated Risk of Adverse Surgical Outcomes Among Patients With Recent COVID-19 Infection Using Target Trial Emulation Methods
Source: JAMA Netw Open. 2023 Mar 28;6(3):e234876. doi: 10.1001/jamanetworkopen.2023.4876 (PMC10051067; doi:10.1001/jamanetworkopen.2023.4876)
Supplement: Supplement 1. — eMethods. [file jamanetwopen-e234876-s001.pdf]

Supplementary Online Content

O'Brien WJ, Gupta K, Itani KMF. Estimated risk of adverse surgical outcomes among patients with recent COVID-19 infection using target trial emulation methods. *JAMA Netw Open.* 2023;6(3):e234876. doi:10.1001/jamanetworkopen.2023.4876

eMethods.

This supplementary material has been provided by the authors to give readers additional information about their work.

© 2023 O'Brien WJ et al. *JAMA Network Open.*

eMethods.

Study Design and Data Sources

This was a retrospective observational database study using the target trial emulation framework. The hypothetical trial is one where preoperative patients randomly acquire Covid-19 infection in the 30 or 60 days prior to surgery, and their postoperative outcomes are compared with a control group that was without Covid-19 infection within the same timeframe. While a randomized control trial (RCT) of this nature is obviously impossible, the emulation approach can reduce certain types of bias in observational studies. The Veterans Affairs Surgical Quality Improvement Program (VASQIP) was the data source for index surgeries, demographic and clinical characteristics, and adverse postoperative outcomes. The VA Covid-19 Shared Data Resource was used to assess vaccination and prior infection status for all participants. Approval was obtained from VA Boston Institutional Review Board, and STROBE guidelines were followed in reporting this study.

Eligibility and Baseline Characteristics

Eligibility criteria was major surgery performed during January 1, 2021 – September 30, 2021 reviewed by the Veterans Affairs Surgical Quality Improvement Program (VASQIP). The start date was chosen to coincide with widespread vaccine availability, as there was likely an overall change in perceptions of safety in operating on vaccinated patients, making comparisons with the previous year difficult. The end date reflects the most recent VASQIP data available. The specialties of anesthesia, endoscopy, ophthalmology, oral, proctology, and transplantation were excluded due to low volume. We obtained data on patient factors that were known on day 60 ("time zero" for pseudo-randomization) prior to the index surgery and were thought to be predictive of allocation to the exposure groups of interest. Demographics included age, race, and sex. Clinical characteristics were American Society of Anesthesiologists Classification, body mass index (BMI), serum albumin, smoking, and diabetes. A 5-level geographical region was included to control for possible differences in SARS-CoV-2 strain prevalence in different parts of the US.

© 2023 O'Brien WJ et al. *JAMA Network Open.*

Vaccination status was defined as a 3-level variable: “fully” if the final dose had been administered at least 14 days before time zero, “partial” if only the 1st in a 2-dose series was administered, otherwise “none.” All VA enrollees were encouraged and eligible to receive vaccines at no cost in order to reduce risk of transmission, severe illness, hospitalization, and death.

Exposures

We defined 3 mutually exclusive exposure groups based on the most recent infection leading up to surgery: infection days 1-30, in days 31-60, or none. Infections prior to day 60 were not assessed. Covid-19 infection was defined as any positive/detected polymerase chain reaction (PCR) or rapid antigen (e.g. Binax) test. National VA policy during the study was to perform universal preoperative screening for Covid-19 infection. The dominant SARS-CoV-2 strains affecting our cohort were Alpha in early 2021, followed by Delta later that summer, both of which were notable for contagiousness and severe disease compared with the original strain. At the time, there were no oral outpatient therapies available, and outpatient use of Remdesivir had not been authorized. Therefore, we did not control for treatments as post-exposure confounders.

Outcomes

Selected 30-day postoperative adverse outcomes were then identified. Infectious outcomes consisted of pneumonia, sepsis, and superficial, deep wound, or organ space surgical site infections (SSI). Respiratory outcomes were reintubation or failure to wean from ventilator. The “any” adverse outcome category consisted of infectious or respiratory outcomes, as well as 30-day mortality, cardiac outcomes (myocardial infarction or cardiac arrest), nervous system outcomes (peripheral nerve injury or coma lasting > 24 hours), and vascular outcomes (pulmonary embolism, graft failure, cerebrovascular accident/stroke, deep vein thrombosis, or bleeding requiring > 4 units packed red blood cells).

© 2023 O’Brien WJ et al. *JAMA Network Open.*

Statistical Analysis

Analyses were performed in R 4.2.1, including the *twang* package. A multinomial propensity score model estimated each participant's probability of allocation to each exposure group. The estimation method was the generalized boosting model (GBM) with 3 levels of interaction and 5000 trees. Inverse probability of treatment weights (IPTW) were applied to balance all characteristics that we observed across exposure groups. IPTW is defined as the reciprocal of the probability of receiving the treatment (in this case, exposure) that was actually received. Weights were stabilized to preserve sample size and avoid the need for standard error adjustment. Balance was assessed using standardized mean difference (SMD), and we decide *a priori* that any weighted covariates with SMD > 0.2 would be included in the outcome model for doubly robust estimation.

Weighted logistic regression estimated the odds ratio (OR) of any outcome as a function of exposure group, perioperative factors (since they were not known at time zero), as well as any covariates that remain imbalanced (i.e. SMD > 0.2) after weighting.

© 2023 O'Brien WJ et al. *JAMA Network Open.*
