# Supplementary material for: Estimated Risk of Adverse Surgical Outcomes Among Patients With Recent COVID-19 Infection Using Target Trial Emulation Methods
Source: JAMA Netw Open. 2023 Mar 28;6(3):e234876. doi: 10.1001/jamanetworkopen.2023.4876 (PMC10051067; doi:10.1001/jamanetworkopen.2023.4876)
Supplement: Supplement 2. — Data Sharing Statement [file jamanetwopen-e234876-s002.pdf]

Data Sharing Statement

O'Brien. Estimated Risk of Adverse Surgical Outcomes Among Patients With Recent COVID-19 Infection Using Target Trial Emulation Methods. *JAMA Netw Open*. Published March 28, 2023. doi:10.1001/jamanetworkopen.2023.4876

Data
Data available: No
